# Drugs and Healthy Aging

**DOI:** 10.1007/s40266-025-01208-2

**Published:** 2025-07-03

**Authors:** Sarah N. Hilmer, Luigi Ferrucci, Antonio Cherubini

**Affiliations:** 1 Kolling Institute, Northern Sydney Local Health District and The University of Sydney, St. Leonards, Australia; 2 Intramural Research Program, National Institute on Aging, Baltimore, MD 21224 USA; 3 Geriatria, Accettazione geriatrica e Centro di ricerca per l’invecchiamento, IRCCS INRCA, Ancona, Italy; 4 Department of Clinical and Molecular Sciences, Università Politecnica delle Marche, Ancona, Italy

## Abstract

Appropriate drug treatment can enhance the likelihood of experiencing healthy aging and maintaining functional ability up to very late in life. Strong evidence exists that overall drugs can help prevent and manage diseases. However, such evidence is mostly available from studies that are not representative of older people and do not include functional/well-being outcomes. Therapeutic drugs can also impair physical and cognitive function and social interactions, particularly in the context of polypharmacy, multimorbidity and frailty. Certain drugs can affect the ability to exercise and consume a healthy diet, which are key nonpharmacological interventions that promote healthy aging. Yet, exercise and nutritional interventions can help manage adverse drug reactions. In the future, drugs (gerotherapeutics) may be developed that slow the aging process, which should prevent or delay the incidence and progression of many chronic diseases, improving healthy aging.

## Key Points

**Table:** 

With the aging of the population internationally, promoting healthy aging, i.e., the maintenance of good function and well-being into old age, is increasingly important.
Therapeutic drugs can improve healthy aging by preventing and managing diseases. Adverse drug reactions may oppose healthy aging by impairing physical, cognitive or social functioning.
Certain drugs interfere with the performance of health behaviors important for healthy aging, such as exercise and nutrition. These health behaviors can help manage adverse drug reactions.
Geroscience research aims to identify drugs that slow the aging process (gerotherapeutics), which may prevent or delay many chronic age-related diseases and promote healthy longevity.

## Introduction

Aging is the most important demographic phenomenon of the twenty-first century. The average life span has increased worldwide, reaching 73 years in 2024, with the greatest rise in high income countries, where it is currently about 81 years. The number of individuals aged 65 years or more has rapidly increased and will reach 800 million worldwide in 2024 [1]. Factors associated with increasing life expectancy include better public health, e.g., better hygiene and nutrition, and progress in both prevention and treatment of common diseases [2].

Current epidemiological data suggest that the positive gain in life expectancy is offset by an expansion of the period of life when people experience diseases and disability, with variability in lifespan and healthspan according to sociodemographic factors [3]. This trend is set to increase the already high global burden of morbidity and multimorbidity in the population. Therefore, expanding health expectancy or “healthspan” has become a priority. This new focus is often referred to as compression of disability and morbidity [4].

**Fig. 1:**
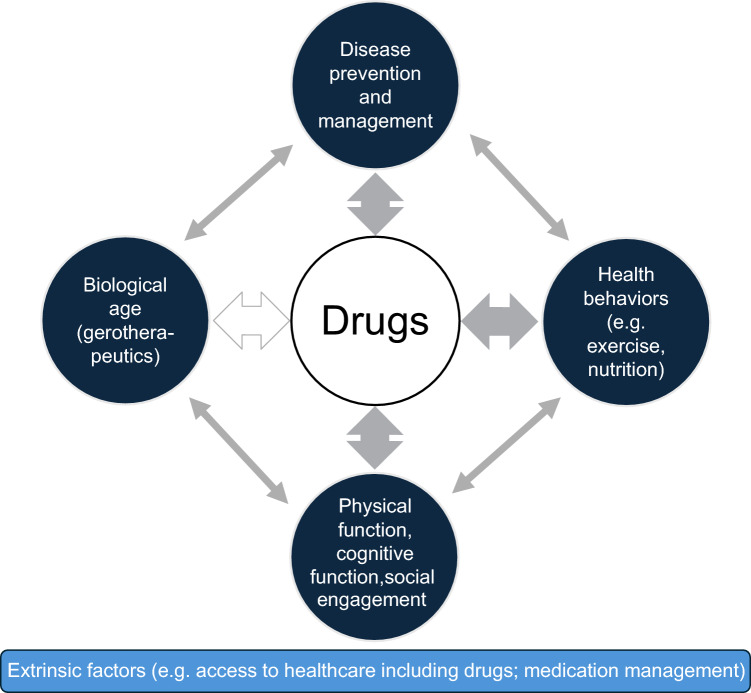
The relationship between drugs and healthy aging. Complex, multidirectional, positive, and negative interactions exist between drugs and factors related to intrinsic capacity (black circles). The arrows between drugs and disease, health behaviors, and function are solid, indicating that these bidirectional relationships are well established. The arrow between biological age and drugs is unfilled because the development of effective gerotherapeutics is a goal that has not currently been realized. The drug factors related to intrinsic capacity are underpinned by factors related to extrinsic capacity (blue rectangle)

While different theoretical constructs of aging have been developed in recent decades [5], the World Health Organization (WHO) 2015 Report on Aging and Health proposed a new definition of healthy aging, i.e., the process of developing and maintaining the functional ability that enables well-being in older age [6]. This new construct recognizes that most older people will develop chronic diseases but, nevertheless, can maintain their functional ability, aiming to live autonomously. The construct involves intrinsic (physical and mental) capacity as well as extrinsic (environmental) capacity. The complex relationship between pharmacotherapy (drugs) and the healthy aging construct is summarized in Fig. 1.

Healthy lifestyle is key in achieving healthy aging, but drug treatment can also play important positive and negative roles. Therapeutic drugs play important roles in optimizing intrinsic capacity. Drugs can help prevent diseases. Drugs can improve disease management, resulting in slower disease progression, less complications, a lower burden of disability, and reduced mortality. The use of drugs in the older population also presents problems that should be carefully considered. First, because participants in clinical trials for drug approval are often younger and healthier than the patients who are prescribed the drugs, there are concerns that the efficacy and safety of drugs are not known in those aged over 80 years and those with multimorbidity, frailty and disability [7, 8]. This might reduce the potential benefits of drugs, not only because it is unknown which patients can benefit and which can be harmed by a specific drug or combination of drugs, but also because the uncertainty concerning the risk–benefit ratio might lead to underprescription of drugs in older patients [9]. Furthermore, drugs can play a direct negative role on physical and cognitive function and on social interactions, hindering the achievement of healthy aging. Indeed, polypharmacy, inappropriate use of drugs, and adverse drug events can contribute to the development or progression of chronic conditions, disability, and frailty [10]. Extrinsic capacity can have an impact on access to healthcare and optimal medication management.

Recent data suggest that the pace of life expectancy increase is slowing [11], challenging the hypothesis that in the next decades almost everyone would reach 100 years [12]. This phenomenon is explained, at least in part, by increased mortality in the middle-aged population, and it is likely that the decline in longevity is accompanied by reduced healthspan. To counteract this dangerous trend, it is tantamount to expand research on methods to increase both life and health expectancy. Accomplishing this goal may require breakthrough discoveries of interventions that can slow down the pace of aging. This approach is consistent with the geroscience hypothesis, posing that aging is the major risk factor for several noncommunicable diseases due to shared biological mechanisms. An important corollary of the geroscience hypothesis is that it should be possible to develop drugs (gerotherapeutics) that can simultaneously slow down the aging process and reduce the occurrence and progression of chronic diseases [13].

## The Role of Therapeutic Drugs in Prevention and Management of Diseases to Improve Healthy Aging

### Drugs and Primary Prevention of Disease

The most effective strategy to maintain intrinsic capacity for healthy aging would be preventing the onset of diseases as people age. Improvements in environmental conditions, lifestyle, and cardiovascular prevention led to the increase in life expectancy and the aging of the population. However, the prevention of other noncommunicable diseases that contribute to disability in old age, such as musculoskeletal diseases, cancers, and neurodegenerative disease, has not been as successful. Interestingly, the reduction in mortality from stroke has been accompanied by an increase in mortality from dementia [14]. This section focuses on drugs to prevent specific diseases, using the examples from cardiovascular disease and cancer. Vaccinations to prevent infections, cancers, or other diseases are out of scope. Section 4 will discuss the role of gerotherapeutics that target aging itself to prevent multiple diseases associated with aging.

Cardiovascular diseases are the principal cause of death and an important cause of morbidity and healthcare use and, therefore, their prevention is a major aim of healthcare. In older adults, cardiovascular diseases are associated with an increased burden of disability and reduced quality of life [15]. Statins are recommended by existing guidelines for their efficacy in primary prevention of myocardial infarction and ischemic stroke, with a reduction in all-cause mortality [16]. While statins are effective in primary prevention of cardiovascular disease [17] and, therefore, promote healthy aging, their effectiveness for this indication in people aged over 75 years remains uncertain [18]. However, the safety of stopping statins is also uncertain [19], and in most older people, statins are well tolerated. Therefore, currently it is difficult to determine how best to use statins in those aged over 75 years to promote healthy aging. Ongoing studies are evaluating the risk–benefit ratio of statin therapy for primary prevention in individuals older than 75 years [15].

Population aging has led to a progressive increase of cancer incidence in old age [20]. Cancer is, therefore, a major cause of morbidity and mortality in advanced age, and the possibility of preventing this condition is actively pursued by researchers [21]. Besides lifestyle and surgical interventions, a few drugs have been demonstrated to be effective in the prevention of some cancers. For example, selective estrogen receptor modulators and aromatase inhibitors are recommended to prevent breast cancer in women who are at high risk of this condition [22].

### Drugs and Disease Management

Advances in healthcare have led to greater survival into old age with diseases, often with multimorbidity. Disease management, involving drug therapy, can contribute to intrinsic capacity and improve healthy aging by slowing disease progression and reducing symptom burden. To realize this potential, the development of therapeutic drugs that are effective and safe in older people with multimorbidity is needed. Aligning with the disease prevention section, this section provides examples from management of cardiovascular disease and cancer.

Secondary prevention of cardiovascular disease is recommended into very old age. Angiotensin receptor blockers, angiotensin-converting enzyme (ACE) inhibitors, angiotensin receptor–neprilysin inhibitors, and sodium-glucose cotransporter 2 inhibitors have been shown to reduce the progression of heart failure, ameliorate symptoms, and prevent its complications [23]. Emerging evidence from subgroup analyses of clinical trials and pharmacovigilance studies supports the efficacy and safety of heart failure medications in frail older people [24–26].

Drug therapy for cancer treatment in older adults contributes to healthy aging. There is growing research on development of drugs that are effective and safe in older adults, as well as optimizing the use of existing chemotherapy in older adults, and the implementation of comprehensive onco-geriatric models of care [27]. For example, emerging treatments for chronic lymphocytic leukemia, which has a median age of onset of 70 years, are orally administered with less interactions and toxicities relevant to older adults [28].

Cancer “survivorship” has gained attention over the past decade, with domains that are highly relevant to healthy aging and frequently involve drugs [29]. Prevention of new cancers can involve chemoprevention, and management of the effects of cancer and its treatment may involve drug therapy, as can management of chronic physical and mental health conditions and health promotion. For instance, endocrine therapy, i.e., treatment with aromatase inhibitors, is recommended in postmenopausal women with hormone receptor-positive breast cancers for 5–10 years to prevent recurrence [30]. Furthermore, patients treated with aromatase inhibitors who are at high risk of fragility fractures should receive osteoporosis therapy [31]. Therefore, drugs can contribute to healthy aging in people after initial cancer treatment, with future opportunities to prevent recurrence further through more personalized medicine and novel therapies.

## The Role of Therapeutic Drugs in Optimizing Physical and Cognitive Function and Social Engagement to Promote Healthy Aging

Optimizing physical and cognitive function and social engagement can be impacted positively or negatively by therapeutic drug use. Medications affect function and engagement through their therapeutic effects and their side effects. The impact of medications on these outcomes differs when drugs are used alone and when they are used in combinations, which is very common in older adults, where polypharmacy is the norm. Therefore, a comprehensive and regularly performed medication review that considers the effects of an older person’s medications on their physical function, cognitive function, and social engagement is critical for promotion of healthy aging. A recent meta-analysis reported moderate certainty evidence that multidomain assessment and management with medication review for older people living at home increased the odds of continuing to live at home and increased independence with instrumental activities of daily living [32]. Access to comprehensive, age-friendly medical care, including medication reviews and medications themselves, is an extrinsic factor that influences healthy aging.

Therapeutic drugs that prevent and manage diseases have great potential to promote healthy aging in older adults, as discussed in Sect. 2. For example, effective management of heart failure reduces shortness of breath, increasing exercise tolerance and opportunities for social engagement. Recently, some clinical trials of heart failure medications have included quality of life as a secondary outcome. A randomized controlled trial of a sodium-glucose cotransporter 2 inhibitor versus placebo, in addition to usual care, in people hospitalized or recently discharged after worsening heart failure, found that treatment improved symptoms, physical function, and quality of life at 4 months [33]. Similarly, in people living with Alzheimer’s dementia, cholinesterase inhibitors (compared with placebo) have been shown to improve cognitive function and some global measures of functional independence [34]. Studies are underway to develop better treatments for dementia prevention and management, targeting the disease processes and symptom control.

However, many drugs impair physical and/or cognitive function in older adults, particularly combinations of drugs with pharmacodynamic or pharmacokinetic interactions, which are common in older people with polypharmacy. Drugs that have anticholinergic or sedative effects, as a therapeutic effect or a side effect, impair cognitive and physical function. Many measures of cumulative exposure to drugs with these effects have been developed and shown in cross sectional and longitudinal observational studies to be associated with functional impairment in older adults. For example, the Drug Burden Index, which is a pharmacological measure of an individual’s total exposure to drugs with anticholinergic and/or sedative effects [35], is associated with a wide range of adverse geriatric outcomes in international observational studies of older adults and interventional preclinical studies [36]. Drugs may impair neurological function through direct actions on neurotransmitters, e.g., antipsychotics or antiemetics causing Parkinsonism; or indirect actions, e.g., antidepressants or diuretics causing hyponatremia, resulting in delirium.

The effects of therapeutic drugs on social engagement are only partly attributable to their effects on physical and cognitive function. Anticholinergic drugs not only impair physical and cognitive function but also cause xerostomia and halitosis, which may limit social engagement. Social engagement is a complex, important outcome that requires careful drug management (i.e., monitoring and titration). For example, diuretics may increase social engagement by improving exercise capacity and physical function but decrease social engagement owing to urinary frequency and incontinence.

There is a two-way relationship between therapeutic drugs and an older person’s participation in nonpharmacological interventions that are important for healthy aging [37]. Drugs can positively or negatively affect exercise tolerance, predominantly through their neurological, cardio-respiratory, musculoskeletal, or endocrine effects. Similarly, drugs that affect taste, appetite, nausea, gastrointestinal absorption, and bowel function can affect nutrition [38]. For example, metformin, which has been repurposed as a potential gerotherapeutic (see Sect. 4) can have side effects of anorexia and weight loss. However, some adverse drug effects can be mitigated by exercise and nutrition. For example, corticosteroids cause sarcopenia and osteoporosis, reducing physical function, which can be addressed through resistance exercises and calcium/vitamin D supplementation; calcium channel blockers cause constipation, which can be ameliorated by aerobic exercise and higher fiber/fluid intake. In some situations, exercise therapy can manage a condition, enabling deprescribing, with consequent resolution of adverse drug effects. For example, chronic musculoskeletal pain may respond to exercise programs, resulting in deprescribing of opioids, reducing sedation, nausea, and constipation, potentially improving nutrition, physical function, cognitive function, and social engagement [39].

## Geroscience and Gerotherapeutics to Improve Healthy Aging

Geroscience and gerotherapeutics may be future keys to healthy aging. The term “geroscience” has already been introduced in this article, but it is essential to define the core principles of the geroscience hypothesis in the context of drug treatments for older adults. The geroscience hypothesis provides a framework for understanding the relationship between aging and age-related diseases, viewing aging as the primary risk factor for many chronic conditions [13]. It posits that by targeting the biological mechanisms of aging, we can extend healthspan—the period of life spent in good health—rather than merely increasing lifespan. By deciphering the biology of aging, researchers aim to develop interventions, known as gerotherapeutics, that may delay the aging process, thereby reducing the incidence of multiple diseases simultaneously.

Recent advancements in composite biomarkers, or “aging clocks,” have significantly enhanced the feasibility of this goal. These biomarkers can predict not only chronological aging but also various health outcomes, including multimorbidity, disability, and mortality [40, 41]. Scientists in the field of geroscience assume that these aging clocks track biological aging rather than chronological aging. Since the pace of aging is the primary driver of disease accumulation—even in subclinical conditions—it should be possible to assess the aging rate in individuals who are entirely disease-free. This assessment could help predict those individuals who are at elevated risk for illnesses and disability owing to accelerated aging. They represent the ideal target population for gerotherapeutic interventions aimed at slowing down the aging process.

From a life course perspective, the interventions within the geroscience framework can be categorized into environmental-behavioral and pharmacological approaches. Given the focus of this article, we will primarily discuss the latter. Pharmacological approaches include repurposed drugs that are already approved for disease prevention or management (including some of the drugs for cardiovascular disease discussed in Sect. 2) and novel compounds. Several biological mechanisms driving aging, referred to as “the hallmarks of aging,” have been identified. The original list proposed by López-Otín in 2013 [42] has been revised multiple times [43] and is expected to evolve further as our understanding of aging improves. Notable mechanisms include cellular senescence, mitochondrial function, and epigenetic changes, all of which are being targeted by specific drug treatments.

For illustrative purposes, we will focus on cellular senescence [44]. A comprehensive discussion of this topic is beyond the scope of this article, so we will provide a brief overview. When a cell encounters severe stress from various sources, it may enter a senescent state. This state is marked by replication arrest, resistance to apoptosis, and the substantial secretion of bioactive molecules known collectively as the senescence-associated secretory phenotype (SASP). Although numerous surface markers and morphological changes have been linked to senescent cells, it has become apparent that no single marker can universally identify all senescent cells.

The accumulation of senescent cells can have detrimental effects on the organism, as these cells lose their original functions. In addition, the SASP can trigger significant inflammation and degenerative changes in neighboring cells and the extracellular matrix. Furthermore, senescent cells often promote the production of more senescent cells, both locally and in distant tissues. Animal studies have demonstrated a correlation between the accumulation of senescent cells and phenotypes and functional limitations typically associated with aging. Importantly, research indicates that removing these cells through various methods can prevent or delay the emergence of characteristics linked to aging.

Drugs developed to counteract the effects of senescent cells fall into two main categories: senolytic and senomorphic agents [45]. Senolytic drugs are thought to eliminate senescent cells by inducing apoptosis, while senomorphic drugs target the production of SASP components. Recently, there has been a strong emphasis on identifying natural compounds and repurposed drugs with potential senolytic or senomorphic properties, leading to the discovery of many promising candidates. For instance, significant work in animal models has involved two repurposed drugs: dasatinib, used to treat specific types of chronic myeloid leukemia, and quercetin, a flavonoid known for its potent antioxidant properties. Many approved medications for various conditions have demonstrated senolytic and/or senomorphic properties in in vitro and in animal studies [45]. However, there is currently insufficient evidence to support the use of any of these compounds as therapeutics for humans.

The testing of gerotherapeutics in clinical trials is still in its early stages, and progress in this field faces many regulatory challenges. Most regulatory agencies require that a drug approved for medical use must demonstrate a significant therapeutic benefit over existing treatments or effectively treat a specific disease or condition, making it difficult to interpret “slowing down aging” or “promoting healthy aging” within this framework. A potential strategy is to design trials that utilize proxy measures of healthy aging as outcomes, such as multimorbidity or disability. However, the necessary sample size and follow-up duration for these trials can be quite costly and unlikely to receive funding through traditional channels. In the context of drug repurposing, an attractive alternative is to incorporate “geriatric outcomes," either biomarker-based or functional-based, into existing trials where drugs are tested for a specific disease indication [46]. While these studies may not be optimally designed for these additional outcomes, they can still yield valuable information to identify promising candidates for further research and the design of future clinical trials.

Active discussions in literature address the special features necessary for clinical trials testing the effectiveness of gerotherapeutics. Here, we outline several essential considerations/recommendations:

- **Maximize inclusion criteria through adaptive design:** Employ an adaptive design to broaden inclusion criteria, facilitating participant recruitment and conducting predefined interim analyses of findings. It is crucial to consider age-related factors affecting older adults, such as comorbidities, polypharmacy, and overall health status. Study protocols should be flexible enough to adapt based on interim results, particularly in instances of low recruitment or variable effectiveness across different demographic groups.
- **Select relevant endpoints for older adults:** Choose endpoints that are meaningful for older individuals, especially those with multimorbidity and frailty. This includes measures such as quality of life, functional independence, cognitive function, and the reduction of falls or hospitalizations. In addition, both biological and phenotypic indicators that could impact the effectiveness of the intervention should be evaluated, as these can inform result interpretation and help refine future trials.
- **Incorporate multimorbidity in study design:** Design studies to encompass a broad spectrum of multimorbidity, allowing for stratified analyses. Given the prevalence of comorbidities in older populations, it is essential to account for these conditions when analyzing results.
- **Enhance eligibility for older adults through safety measures**: Expand eligibility criteria for older individuals with multimorbidity and frailty by integrating safety protocols. Strategies should aim to optimize participant involvement while minimizing side effects and adverse events. Potential measures could include personalized support and the use of digital media or wearable devices to monitor participant health.
- **Utilize proxy assessments:** Incorporate assessments from caregivers and family members, as their insights can provide valuable information regarding the patient’s health status and treatment effects.
- **Allow for extended follow-up in gerotherapeutic trials:** Recognize that gerotherapeutic trials may require prolonged follow-up periods to thoroughly evaluate both the positive and negative effects of interventions over time.
- **Emphasize multiple levels of measurement:** Employ a variety of biological, phenotypic, and functional assessments to gain a comprehensive understanding of the causal pathways that contribute to the success or failure of achieving desired outcomes.

Many of these recommendations overlap with recent international guiding principles for evaluation of therapeutic drugs for older people [7, 8]. Lessons from geriatric pharmacology apply to the use of gerotherapeutics in research and to their use in clinical practice, with older people increasingly taking self-initiated potential gerotherapeutics. Polypharmacy and multimorbidity are common in older adults, and, therefore, drug–drug and drug–disease interactions must be considered, which may involve the potential gerotherapeutic. Adverse drug reactions in older adults can present as nonspecific geriatric syndromes, such as falls or delirium, and a high index of suspicion is needed to detect these in clinical trials and clinical practice. More than half of older adults take complementary medicines, which are rarely disclosed to their treating clinicians. In research and practice, it is important to ask older patients whether they are taking any potential gerotherapeutics, which may not be prescribed and may cause adverse reactions and interactions.

## Conclusions

There are opportunities to better align drug use and future drug development with healthy aging. Comprehensive medication review for older adults must consider the impact of all of the person’s medications on all aspects of healthy aging, acknowledging the complex, two-way relationships highlighted above. This requires frequent, careful monitoring of specific and global outcomes relevant to healthy aging and adjustment of medications, understanding the person’s medication use (including adherence), diagnoses, function and goals. It is important to consider the possibilities of undertreatment and overtreatment and interactions of therapeutic drugs with other drugs, including with potential gerotherapeutics, with diseases, with aging physiology, with exercise, and with nutrition. Future drug development opportunities [8] include representative recruitment [47], healthy aging outcomes in trials of therapeutic drugs designed to prevent or treat diseases [46], and development or repurposing of drugs that specifically target healthy aging (gerotherapeutics) [13].
